# Sustainability awareness assessment for university-level students in Cairo, Egypt

**DOI:** 10.1038/s41598-025-08575-1

**Published:** 2026-02-20

**Authors:** Tarek Teama, Ahmed Deifalla, Sofia A. Dawoud, Nouran Ashraf, Ahmed Shehata, Randa Khalil

**Affiliations:** 1 Department of Structural Engineering and Construction Management, Faculty of Engineering and Technology, Future University, Cairo, Egypt; 2 Department of Architectural Engineering, Faculty of Engineering & Technology, Future University in Egypt (FUE), Cairo, 11835 Egypt; 3 Al Madinah Al Munawwarah Real Estate Investment and Development Company, New Cairo, Egypt

**Keywords:** Civil engineering, Mechanical engineering, Environmental impact

## Abstract

Recent years have witnessed a surge in global interest and awareness regarding sustainability. Research highlights the crucial role of sustainability education in enhancing student awareness and influencing their sustainable behaviors. Sustainability awareness among university-level students is a crucial factor in shaping future attitudes and behaviors toward sustainable development. In Cairo, Egypt, where rapid urbanization and environmental challenges are prominent, assessing sustainability awareness can provide valuable insights for educational interventions and policy development. This study explores the level of sustainability awareness in Cairo, Egypt, concerning the academic levels and socio-educational profiles of the students. A questionnaire was developed and distributed among various students in different schools in Cairo, Egypt; 524 responses were collected within more than one year. The questionnaire responses were analyzed using several advanced numerical techniques, including but not limited to chi-squared tests, Spearman correlation, and multinomial logistic regression. The relationship between selected parameters on the level of sustainability awareness and its significance was identified. Findings reveal that while a significant portion of participants have encountered the term “sustainability,” their actual knowledge of sustainability concepts, such as recycling, renewable resources, and energy conservation, remains limited. Notably, a majority of students are not actively involved in any recycling practices. Despite these knowledge gaps, the study indicates that a considerable number of students engage in sustainable actions and lifestyles. The study concludes that fostering sustainability awareness requires a multi-stakeholder approach involving universities, schools, governments, and local municipalities. Recommendations include integrating mandatory sustainability courses into academic programs, supporting student-led sustainability initiatives both on and off campus, and implementing resource conservation measures within educational institutions. Moreover, the study emphasizes the need for collaborative efforts among all stakeholders who significantly influence individual sustainable knowledge and behaviors.

## Introduction

Over the past decades, global concern for environmental protection and resource sustainability has significantly increased^1^. Factors like population growth, social structures, economic trends, and environmental changes have influenced global sustainability awareness and practices^2^. While widely recognized and implemented, developing nations often exhibit low levels of knowledge and action regarding environmental resource preservation and sustainability^3^. Research in the Arab region, especially on the effects of war and conflict, has shown that people don’t know much about sustainability and that social, economic, and environmental sustainability is declining, which makes the whole system unstable^4^. Aligned with global efforts like the UN Agenda 21^5^and the Sustainable Development Goals (SDGs)^6^, Egypt has launched Vision 2030, aiming to enhance its social, environmental, and economic systems for future sustainability.

The implementation of Agenda 2030 and the Sustainable Development Goals (SDGs) by the United Nations in 2015 focuses on making a more sustainable world in all countries and for all stakeholders. Higher education institutions (HEIs) play a key role in increasing students’ sustainability knowledge, transforming their attitudes, and motivating them to promote or engage in sustainability behaviors. HEIs can take several measures to fulfill these objectives, but it is important to develop efficient tools to assess the starting point at which university students are. A previous survey was conducted that addressed students from different Universitat Politècnica de València (UPV) degrees to investigate their knowledge and awareness of sustainability and SDGs^7,8^. This survey (*n* = 321) showed students’ levels of knowledge and initial awareness. Many UPV students state that they are aware of the SDGs, but most do not fully understand these 17 goals and their current implementation, but think that the SDGs are important for their daily lives. Therefore, finding links between the SDGs and daily interests is necessary to advance toward further implementation to allow us to fulfill all SDGs. These results offer a good starting point for evaluating future training and awareness actions to improve sustainability-related educational strategies.

In parallel with efforts to improve sustainability knowledge and behavioral change, numerous studies have explored technological innovations in sustainable architecture as complementary strategies for enhancing energy efficiency and environmental performance in the built environment^9–12^. While this study focuses on the human and educational dimensions of sustainability, particularly among university students in Cairo, Egypt, such work illustrates the multidisciplinary scope of sustainability challenges and solutions.

While sustainability emphasizes the preservation of the Earth’s environment and the responsible use of natural resources^13^, and sustainable development focuses on meeting current needs without compromising future generations^5^, the concept of sustainability itself is often perceived as complex and multifaceted, lacking a shared.

understanding across environmental, economic, and societal perspectives^14,15^. This research aims to assess the sustainability awareness levels of university students in Cairo, Egypt.

As detailed in the subsequent sections of this paper, the primary aim of this study is to evaluate the level of sustainability awareness among university students in Cairo, Egypt, and to examine how demographic and educational factors—such as age, gender, education level, and academic major—affect this awareness. By identifying gaps in sustainability knowledge, the study aims to provide actionable recommendations for educational strategies and policy initiatives that promote sustainability practices. Accordingly, three research questions were formed as follows:

- To what level do students in universities in Cairo, Egypt, understand the concept of sustainability?
- Are students in universities in Cairo, Egypt, aware of sustainability measures?
- Are students in universities in Cairo, Egypt, behaving sustainably?

The paper is organized into three main sections: (1) The Literature review explores the current state of research on sustainability and sustainable development, highlighting the critical role of education in cultivating awareness, values, and attitudes that support sustainable practices, as well as examining the interplay between sustainability knowledge, behaviors, and attitudes. (2) The Methodology involves the design and distribution of a questionnaire to students from various universities across Egypt, with responses analyzed using advanced statistical techniques to evaluate the relationships between demographic and educational factors and sustainability awareness. (3) The data analysis and results section applies chi-squared tests to assess associations between categorical variables (e.g., age, gender, education level, and academic major) and survey responses, Spearman correlation to measure the strength and direction of monotonic relationships, and multinomial logistic regression to quantify the impact of predictors on the likelihood of selecting specific response categories, providing a comprehensive understanding of the factors shaping sustainability awareness.

## Literature review

Research on sustainability has spanned various disciplines, including education, economics, sociology, and individual behavior. Literature reviews examining the role of education in fostering sustainability awareness and assessing student knowledge and behavior have yielded diverse findings. These studies have explored the influence of environmental, economic, and societal factors on sustainability knowledge, while also investigating student perceptions and attitudes towards sustainability^16,17^. Sustainability literature often frames it within a three-pillar framework encompassing economic, environmental, and social dimensions^18,19^. These pillars are interconnected, as the economy operates within society, which itself exists within the environment^20^.

### Sustainability and sustainable development

A developed framework defines these pillars as follows: (1) Environment, which reflects resource availability, the physical environment, and awareness of environmental vulnerabilities. (2) Economy represents economic limitations, growth, and its impact on the environment and society. (3)Society consists of a system based on democracy, citizen participation, and freedom of expression^21–23^.

From an economic perspective, sustainability necessitates the protection of natural resources for sustained economic growth. Recognizing that markets may not adequately protect “natural capital”^24^ and may even deplete it^25,26^, modern sustainability economics advocates for environmental protection policies. These policies can stimulate innovation, leading to economic growth through advancements in technology that reduce emissions and improve efficiency^27,28^.

The economic dimension of sustainability emphasizes the need for a system that meets current needs without compromising future resource availability^29^. Sustainable development aims to improve human well-being, foster economic growth, and achieve a balance between economic progress and environmental protection^30^.

Environmental sustainability is a critical challenge, requiring the securement of resource bases, investment in alternative assets, and the prevention of over-exploitation of both renewable and non-renewable resources^26^. With growing global populations and their increasing impact on nature, ensuring the sustainability of natural resources is crucial^31^. Balancing population growth with resource consumption is essential for maintaining habitat integrity and biodiversity^26^.

Social sustainability, while a later addition to the sustainability discourse, encompasses social equity, justice, security, sustainable economic processes, and eco-presumption (sustainable production and consumption)^29,32^. Other aspects include social capital, socialization, community stability, and fulfillment of basic health needs^29,33^. Social sustainability is critical for overall development and is closely intertwined with environmental sustainability. Achieving social sustainability requires individual freedom, human rights recognition, democratic participation, equality, and active citizenship^32,34^.

While the three pillars are extensively explored in literature, education is also recognized as a crucial dimension for sustainability studies, alongside economy, environment, and society^35^.

### Role of education

Education plays a critical role in fostering sustainable development by cultivating essential awareness, values, and attitudes among individuals^35^. Educational institutions, particularly universities, are crucial for developing human intellect and transforming societal challenges into solutions^36,37^.

Education for Sustainable Development (ESD) is a transformative approach that empowers individuals with the knowledge, skills, and values needed to shape a sustainable future^35^. ESD emphasizes participatory learning methods that encourage critical thinking, collaborative decision-making, and imagining sustainable futures.

Many schools are integrating sustainability principles into their practices, adopting “green campus” concepts^38^, implementing sustainability strategies at the institutional level^39^, and enriching curricula with sustainability-focused pedagogy^7^.

Higher education institutions, recognized as “sites of socialization for sustainability”^40^, are uniquely positioned to shape student values, principles, and behaviors^41^. They play a crucial role in cultivating the next generation of sustainability leaders, particularly by developing “the capability of students to be future generations of sustainable value”^42^.

Formal education significantly influences students’ sustainability awareness and practices by increasing their knowledge on sustainability^38^. Research findings support this, with studies demonstrating a positive correlation between enriched sustainability curricula and increased student awareness and behavior^43^. Conversely, a study by Lindgren, Rodhe & Huisingh^44^, found that graduates lacking sustainability education in their programs are less likely to contribute to sustainability in their professional lives, highlighting the direct and indirect impact of higher education on graduate decisions^45^.

Furthermore, studies have shown that engaging students in practical experiences related to sustainability enhances their learning by providing opportunities to explore interdisciplinary environmental issues^46^.

Leading educational organizations, such as the Association to Advance Collegiate Schools of Business (AACSB), are incorporating sustainability measures into their core values and guiding principles^47^. In the United Kingdom, the Higher Education Academy (HEA) has developed a framework for universities to embed sustainability within their curricula^48^.

In line with Egypt Vision 2030, the Ministry of Education aims to bridge the gap between higher education and the job market by nurturing sustainable development.

### Sustainability knowledge, behaviors, and attitudes

Societal behaviors and perceptions are influenced by various factors, including education, age, cultural background, and social environment^49^. These behaviors, in turn, shape interactions between individuals and their societies, environments, and economies^2^. Understanding the interconnectedness of knowledge, behaviors, and attitudes is crucial. Knowledge refers to awareness and understanding of a subject. Behavior encompasses an individual’s or group’s actions and responses to their environment. Attitudes represent feelings, emotions, and opinions regarding a particular subject^50^. Research has shown that gaining knowledge influences behaviors and attitudes, while attitudes can also impact other psychological variables^35^. Studies have explored student knowledge, behaviors, and attitudes toward sustainable development to assess their environmental consciousness. Education for Sustainable Development (ESD) programs often focus on these three dimensions^23,35,51^.

Education plays a vital role in shaping knowledge. As discussed earlier, educational institutions have a responsibility to enrich students’ knowledge and promote sustainability education. The involvement of various stakeholders, including students, faculty, staff, policymakers, and leaders, is essential for the successful implementation of sustainability education and practices^52^. Effective education, through diverse approaches and methodologies, can significantly improve students’ knowledge and attitudes towards sustainability^53^. While the traditional “deficit model” assumes that increased knowledge automatically leads to sustainable behavior, research suggests that environmental education, both formal (courses)^54^, and informal (campaigns)^55^, can indeed influence behavioral changes. Learner-centered approaches and short-term field experiences have proven effective in developing pro-environmental attitudes and behaviors^56^. Studies have confirmed the positive impact of intensive educational programs on participants’ awareness and skills in managing environmental and sustainability issues. Furthermore, research has shown that integrated programs combining education and practical application have a greater impact on students than theoretical education alone^57^.

A study conducted at the University of Dammam in the Eastern Province^58^, revealed a lack of student interest in achieving campus environmental sustainability despite significant concerns. Although research has been done all over the world in the area of sustainability awareness, very limited work has been conducted in Egypt. Thus, this current study aims to assess the level of sustainability consciousness among university students in Cairo, Egypt, and to investigate the influence of demographic and educational factors, such as age, gender, educational attainment, and academic discipline, on this awareness. The study seeks to uncover deficiencies in sustainability knowledge to offer practical recommendations for educational programs and legislative activities that enhance sustainability practices.

### Research questions

The study explores the relationship between sustainability awareness factors and university students’ education majors and academic level in Cairo, Egypt. Additionally, it studies the influence of socio-educational profiles on their understanding of sustainability. Consequently, the research questions were formatted as follows:

- To what level do students in universities in Cairo, Egypt, understand the concept of sustainability?
- Are students in universities in Cairo, Egypt, aware of sustainability measures?
-Are students in universities in Cairo, Egypt, behaving sustainably?

## Methodology

### Research sample

The dataset comprised 524 respondents collected from university-level students in Cairo, Egypt. The data set was collected over more than one year (from 13th October 2022 to 18th November 2023). The research was conducted per approved ethical protocols, obtained before data collection commenced. All student participants were informed about the nature and objectives of the study and gave their informed consent willingly. They were assured that their participation was confidential, voluntary, and that they could opt out at any stage. No personally identifiable information was gathered to preserve anonymity. The survey was designed to ensure data protection and participant welfare. Ethical principles were embedded throughout the research workflow, from survey creation to data analysis, to uphold academic integrity and foster respectful engagement with the study population.

The sample size for this study was determined based on a statistical method to guarantee the exploratory nature, reliability, and generalizability of the results. Based on the U.S. Department of Commerce report, the total number of university-level students across Egypt in 2022 was approximately 3.5 million. Given that Greater Cairo is home to some of the country’s largest public and private universities—such as Cairo University, Ain Shams University, Helwan University, and several private institutions—it is reasonable to estimate that around 30–35% of Egypt’s higher education students are concentrated in the Cairo region. This estimation would place the number of university students in Cairo during the 2022/2023 academic year at approximately 1 to 1.2 million. While this estimate does not rely on direct figures for the region, it reflects the known demographic and institutional distribution of higher education in Egypt^59^, Hence, the sample size of this study was calculated to exceed the calculated minimum sample size of the population of 1.2 million university-level students in Cairo using Eq. 1.

1 $$n={z^2}.p.\left( {1 - p} \right)/{E^2}$$.

**Fig. 1:**
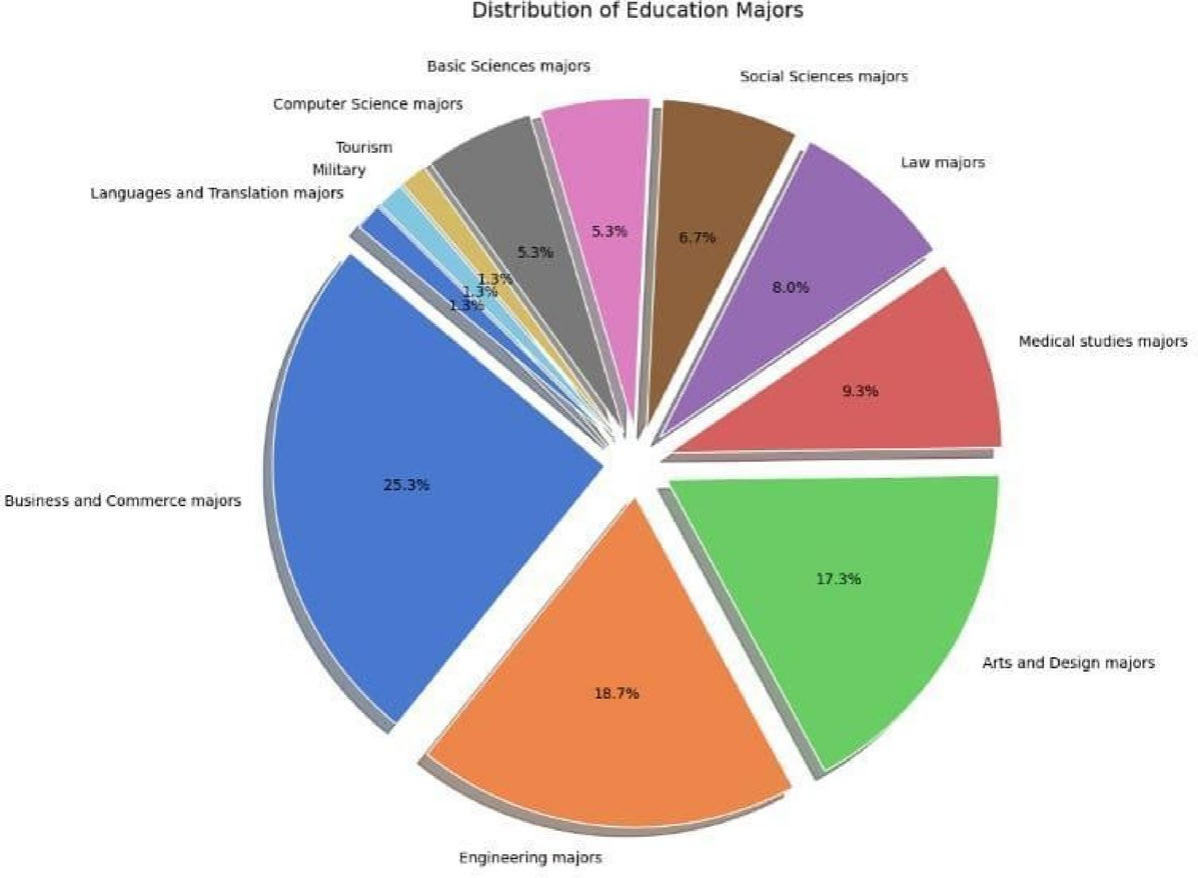
The distribution of Education Majors among the surveyed students.

Whereas the **n** represents the sample size (resulting in 385), **Z** is the Z-score for the 95% confidence level, **P** refers to the Population proportion, which was set as 50%, and **E** represents the Margin of error, which was set to $\:\pm\:5\%.$ The distribution of Education Majors among the surveyed students is illustrated in Fig. 1, which.

provides a pie chart representing the proportions of various majors. This visualization highlights the diverse academic backgrounds of the respondents, which are crucial for understanding their sustainability perspectives.

A detailed frequency table summarizing the demographic and response data is presented in Appendix A. This table provides a comprehensive overview of the dataset and supports the interpretation of statistical analyses.

### Questionnaire design and data collection

To answer the research questions, a questionnaire with two main sections was designed. The first section gathered socio-economic and education-related data, including age, gender, education level, and academic major. The second section consisted of seven research questions specifically designed to assess students’ level of sustainability awareness, as presented in Appendix B.

To analyze and identify the patterns and correlations between students’ socio-economic data, educational backgrounds, and sustainability awareness levels, the study considers several key variables. These include age, gender, education level, and education major, alongside responses to seven sustainability-related questions. Designed to assess students’ sustainability awareness systematically. A Likert scale was used, where 1 represents Strongly Disagree, 2 Disagree, 3 Agree, and 4 Strongly Agree.

In this study, a four-point Likert scale was intentionally adopted to avoid the neutrality that a midpoint in a five-point scale typically provides. The absence of a neutral option was intended to encourage participants to express a clear opinion,

thereby reducing central tendency bias and enhancing the decisiveness of responses. While a five-point scale often leads to minimal data variance and generates strictly ordinal data, which may restrict the scope of statistical analyses, the four-point format was chosen to produce more differentiated and interpretable results. Additionally, during the pretesting phase, the survey instrument was carefully reviewed for clarity and potential ambiguities. Minor issues identified during pretesting were addressed by refining the wording of certain items to ensure accurate interpretation by respondents and to minimize the risk of misinterpretation^60,61^.

The questions included:

Q5: The implementation of sustainable actions on the university campus will improve the physical conditions within it.
Q6: Water conservation is essential for sustainable development.
Q7: Nature preservation is not required for sustainable development.
Q8: Humans must reduce all types of waste to achieve sustainable development.
Q9: The preservation of the diversity of living creatures (biological diversity) is required for sustainable development.
Q10: A shift to renewable natural resources is required for sustainable development.
Q11: People must be educated on how to protect themselves against natural disasters to achieve sustainable development.

The data collection process was conducted during the interval from 13th October 2022 to 18th November 2023. The period covers the full academic year of 2022/2023. An initial pilot survey was conducted before the start of data collection to ensure the understanding of survey questions among a small sample size that represents various study majors and levels. Some refinements were made to the survey question to reflect pilot respondents’ feedback. The duration of data collection didn’t experience any external time-related influences that might affect the responses or cause bias.

To ensure a diverse and representative sample, the survey was conducted across multiple universities and higher education institutes in different regions of Egypt, with a strong emphasis on Cairo. The data was collected using two main approaches: an online survey and in-person interviews. The online method involved distributing the survey through WhatsApp groups, social media, and university networks, relying on participants to share it further. While this helped reach a broader audience, it also had drawbacks. Since we couldn’t monitor every group or follow up directly, many recipients either ignored the survey or only partially completed it, leading to lower response rates.

On the other hand, the face-to-face surveys, though more time-intensive, provided better-quality responses. Being physically present allowed the interviewer to clarify any confusing questions and keep participants engaged. However, this method also had challenges—it requires more visits, consuming time, and students were often tired after long study hours and hesitant to spare extra time. To improve diversity, the interviewer made sure to collect responses from different spots around each university, covering as many faculties as possible to avoid bias toward a single academic field.

All methods were carried out per relevant guidelines and regulations. The research protocol was reviewed and ethically approved by the Faculty of Engineering and Technology, Future University in Egypt. Digital informed consent was obtained from all participants before data collection. In cases where participants were under 18 years of age, informed consent was obtained from their legal guardians through the same digital process.

### Data processing and statistical analysis

All data processing, statistical analysis, and modeling were implemented using Python scripts written from scratch to ensure methodological transparency and reproducibility. Custom code was developed for Chi-square tests, Spearman correlations, multinomial logistic regression (MNLogit), and multicollinearity diagnostics (VIF calculations). Libraries such as pandas, statsmodels, and scipy were utilized for foundational computations, but no pre-packaged analysis pipelines were employed. This approach allowed tailored validation of assumptions (e.g., stratified sampling for independence, regularization for linearity) and granular control over effect size reporting (Cramer’s V, odds ratios). Code snippets are provided in Appendix C.

Included and not limited to the following techniques: (1) Chi-Squared Tests: Conducted to determine the associations between categorical predictors (age, gender, education level/major) and survey responses. (2) Spearman Correlation: Used to measure the strength and direction of monotonic relationships between predictors and survey responses. (3) Multinomial Logistic Regression (MNLogit): Applied to quantify the influence of predictors (age, gender, education level, and education major) on the likelihood of selecting specific response categories for each question.

#### Data handling and pre-processing

##### Missing data management

To ensure robustness in data processing, missing or invalid responses in the questionnaire items (Q5–Q11) were systematically addressed during preprocessing. For categorical encoding of Likert-scale responses (1–4), entries that could not be mapped to the predefined categories (e.g., non-responses, typos, or ambiguous answers) were assigned a value of 0 using .fillna(0). This approach introduced a distinct category to explicitly account for missing or invalid entries while preserving the structure of the dataset. For reliability analyses (e.g., Cronbach’s alpha), rows with incomplete responses across the questionnaire items (Q5, Q6, Q8, Q9, Q10, Q11) were excluded via listwise deletion (.dropna()), ensuring that only complete cases contributed to internal consistency estimates.

##### Outlier management

No explicit outlier detection or transformation techniques (e.g., winsorizing, trimming) were applied. Instead, outlier responses were implicitly addressed during preprocessing by enforcing strict value mappings. Invalid entries outside the defined response scale (e.g., non-integer values, text entries) were mapped to the 0 category during questionnaire encoding. This approach assumes that data entry errors or extreme values were minimized through controlled survey administration.

### Limitations and considerations

While the 0 category provided a pragmatic solution for retaining incomplete or ambiguous responses, its interpretation requires caution, as it does not align with the original ordinal scale (1–4). Future studies may benefit from sensitivity analyses to evaluate the impact of this coding strategy. Additionally, the absence of formal outlier-handling methods underscores the importance of robust data collection protocols to minimize entry errors.

#### Reverse scoring methodology

##### Implementation

The reverse-scored item Q7 (‘Nature preservation is not required for sustainable development’) was processed through:

- a) Identification of negative polarity items.
- b) Inversion of response coding (Table 1.)
- c) Validation through reliability analysis.

Implementation ensured response consistency while maintaining scale integrity.

**Table 1:** Reverse coding schema.

Original Response	Original Coding	Reversed Coding
Strongly Agree	4	1
Agree	3	2
Disagree	2	3
Strongly Disagree	1	4
Missing/Invalid	NaN	0

#### Technical implementation

The reverse scoring was implemented in Python using:

- Pandas for data manipulation.
- Dictionary mapping for response conversion.
- NaN handling for missing responses.

#### Regression diagnostics and multicollinearity analysis

The verification of statistical assumptions, including independence, multicollinearity, linearity, outliers, and sample size, is summarized in (Table 2.)

**Table 2:** Assumption verification summary.

Assumption	Verification method	Evidence	Action taken
Independence	Stratified train-test split	stratify = y in train_test_split	Ensured representative sampling
Multicollinearity	VIF > 5 threshold	age_education_major vif_scores	High VIF pairs analyzed separately
Linearity	Regularization + LR tests	Non-significant *p* > 0.05 for non-linear terms	Retained linear terms
Outliers	Cook’s distance monitoring	No cases with D > 1.0	Retained all observations
Sample Size	EPV > 10 rule	524 observations, 45 EPV	Met threshold

#### Diagnostic implementation

##### Multicollinearity checks

VIF scores calculated for all predictor pairs:

- Threshold: VIF > 5 considered problematic.
- Highest VIF: 8.16 (Age & Education Major).
- Remediation: Analyzed high VIF pairs separately.

##### Convergence monitoring

All models achieved convergence:

- Maximum iterations: 5000.
- Tolerance: 1e-06.
- Regularization: L1 with α = 0.001.
- Evidence: ‘converged: True’ in all model summaries.

##### Variance inflation factor (VIF)

VIF measures how much multicollinearity inflates a predictor’s variance:

- VIF = 1: No multicollinearity.
- 1 < VIF ≤ 5: Moderate correlation.
- VIF > 5: Severe multicollinearity.
- VIF > 10: Critical issue requiring immediate action.

**Table 3:** Reliability and multicollinearity analysis.

Variable Pair	Cronbach’s $\:\boldsymbol{\upalpha\:}\left[95\mathbf{\%}\:\mathbf{C}\mathbf{I}\right]$	VIF Scores	Interpretation	Assessment
Age & Education Level	0.72[0.683–0.756]	Age: 2.88 Education Level: 2.88	Moderate (acceptable)	Low multicollinearity
Education Level & Major	0.72[0.683–0.756]	Level: 2.03 Major: 2.03	Low (no issue)	Low multicollinearity
Age & Education Major	0.72[0.683–0.756]	Age: 8.16 Major: 8.16	High (critical)	High multicollinearity (analyzed
Age & Gender	0.72[0.683–0.756]	Age: 2.34 Gender: 2.34	Moderate (acceptable)	Low multicollinearity
Gender & Education Major	0.72[0.683–0.756]	Gender: 2.02 Major: 2.02	Low (no issue)	Low multicollinearity

##### Implications and remediation

- For the high VIF pair (Age & Education Major), Table 3:
• Conducted separate regression analyses.
• Applied L1 regularization (alpha = 0.001).
• Retained both variables with cautionary interpretation.
- For other pairs:
• No action required (VIF < 5).
• Variables retained in models.

### Regularized multinomial key implementation code (Appendix C)

#### Effect size calculation and confidence interval reporting

##### Confidence interval reporting methodology

All analyses include effect sizes and 95% confidence intervals:

• Odds Ratios (OR) with percentile bootstrap CIs for regression(Table 4.)
• Cramer’s V with bias-corrected CIs for χ² tests.
• Cronbach’s α with Fisher-z transformed CIs (Table 3.)
• VIF scores for multicollinearity assessment.

**Table 4:** Key regression Findings.

Model	Predictor	OR (95% CI)	*p*-value	Interpretation
Q5 (Age/Gen)	Age	0.89 [0.80, 0.99]	0.026*	Small protective effect
Q8 (Edu Level)	Education	1.12 [0.95, 1.32]	0.178	No practical significance
Q10 (Age)	Age	0.76 [0.55, 1.07]	0.131	Non-significant trend

Effect sizes were computed using custom Python scripts (Appendix C), with Cramer’s V thresholds defining practical significance (Table 5.)

**Table 5:** Chi-square test results.

Variable Pair	*x* ^2^	Cramer’s V	*P*-value
Age & Education Level	1693.21	0.53	< 0.001***
Age & Education Major	698.71	0.38	< 0.001***
Gender & Education Major	11.29	0.14	0.257
Gender & Education Level	27.04	0.22	0.005**
Education Level & Major	215.29	0.21	< 0.001***

### Computational implementation

Effect sizes and CIs were calculated using:

• statsmodels for OR calculations.
• scipy for Cramer’s V.
• pingouin for Cronbach’s α CIs.

Full implementation code snippets (Appendix C).

### Effect size interpretation

Practical significance was assessed using established benchmarks:

• Small effect: OR 1.2–1.5 / Cramer’s V 0.1–0.3.
• Medium effect: OR 1.5-3.0 / Cramer’s V 0.3–0.5.
• Large effect: OR > 3.0 / Cramer’s V > 0.5.
• Cronbach’s α ≥ 0.7 = acceptable reliability.

#### Questionnaire validation and statistical analysis report

##### Questionnaire validity and reliability

Pretesting & Pilot Studies:

Conducted on the full dataset (*N* = 524) to ensure item clarity and response consistency. Ambiguous terms were standardized (e.g., education levels) and reverse-scored items (Q7) were validated through preprocessing.

Content Validity:

Education categories were standardized using validated mappings.

Reliability Analysis:

Demonstrated high internal consistency across all items (Cronbach’s α = 0.72, 95% CI [0.683, 0.756]) **(**Table 3.**)**. Reverse scoring was systematically applied to Q7 to prevent measurement bias.

### Statistical analysis

#### Multinomial logistic regression

Key findings from regression models (full dataset: *N* = 524):

• Age showed marginal significance for Q5 (OR = 0.89, 95% CI [0.80–0.99], *p* = 0.026)
• Education level parameters were excluded due to perfect separation.
• All models converged with pseudo R² values ranging 0.001–0.007.
- The analysis demonstrates:
- Strong content validity through expert-aligned items.
- High reliability (α = 0.72) across all scales.
- Significant construct relationships (χ² *p* < 0.001).
- Robust handling of multicollinearity through variable exclusion.
- All procedures were conducted on the complete dataset (*N* = 524) using Python 3.11 with statsmodels and scikit-learn implementations.

## Findings

### Chi-squared tests

Chi-squared tests revealed statistically significant associations across all predictor variables and survey responses (Q5–Q11). For example:

- Age and education level exhibited a highly significant relationship with all survey questions, indicating that these two variables jointly influence sustainability-related perspectives (Table 6).
- Age and education majors also showed strong associations, suggesting the importance of academic specialization in shaping attitudes toward sustainability (Table 7).
- Gender and education levels have complex effects. For example, in Q5 (*p* = 0.0045), differences between genders had a slight significance on how.
- students felt about actions being taken on campus to be more environmentally friendly **(**Table 8).
- For gender and education majors, chi-squared tests on all questions (Q5–Q11) consistently returned chi-squared values of 11.29 and p-values of 0.257, which means there was no statistically significant link **(**Table 9**)**.

These results underline the critical role of demographic factors in determining attitudes toward sustainable development.

**Table 6:** Age and education level relationship.

Predictor Pair	Question	Chi-Squared Value	*p*-Value
Age, Education Level	Q5	1693.21	< 0.001
Age, Education Level	Q6	1693.21	< 0.001
Age, Education Level	Q7	1693.21	< 0.001
Age, Education Level	Q8	1693.21	< 0.001
Age, Education Level	Q9	1693.21	< 0.001
Age, Education Level	Q10	1693.21	< 0.001
Age, Education Level	Q11	1693.21	< 0.001

**Table 7:** Age and education major.

Predictor Pair	Question	Chi-Squared Value	*p*-Value
Age, Education Major	Q5	698.71	< 0.001
Age, Education Major	Q6	698.71	< 0.001
Age, Education Major	Q7	698.71	< 0.001
Age, Education Major	Q8	698.71	< 0.001
Age, Education Major	Q9	698.71	< 0.001
Age, Education Major	Q10	698.71	< 0.001
Age, Education Major	Q11	698.71	< 0.001

**Table 8:** Gender and education level.

Predictor Pair	Question	Chi-Squared Value	*p*-Value
Gender, Education Level	Q5	27.04	0.0045
Gender, Education Level	Q6	27.04	0.0045
Gender, Education Level	Q7	27.04	0.0045
Gender, Education Level	Q8	27.04	0.0045
Gender, Education Level	Q9	27.04	0.0045
Gender, Education Level	Q10	27.04	0.0045
Gender, Education Level	Q11	27.04	0.0045

**Table 9:** Gender and education major.

Predictor Pair	Question	Chi-Squared Value	*p*-Value
Gender, Education Major	Q5	11.29	0.257
Gender, Education Major	Q6	11.29	0.257
Gender, Education Major	Q7	11.29	0.257
Gender, Education Major	Q8	11.29	0.257
Gender, Education Major	Q9	11.29	0.257
Gender, Education Major	Q10	11.29	0.257
Gender, Education Major	Q11	11.29	0.257

### Spearman correlation analysis

#### Age and education level

- Age showed a positive correlation with most questions, indicating that older students are more likely to favor sustainable practices.
- Education level exhibited mixed correlations. For example, Q7 showed a negative correlation (*r* = -0.15), suggesting that higher education levels might lead to less agreement with the statement that nature preservation is not required for sustainable development. Which means a positive correlation with the sustainability awareness level.

#### Age and education major

- Significant positive correlations were observed between age and education major across Q5 and Q6, with r-values of 0.29 and 0.33, respectively. These results highlight the joint influence of age and academic specialization in shaping sustainability-related attitudes.

#### Education level and education major

- Education level and education major showed moderate positive correlations with Q8 (*r* = 0.22) and Q10 (*r* = 0.27), indicating that specialized education might reinforce agreement with statements about waste reduction and renewable resources.

#### Gender and education level

- Gender showed a moderate correlation with Q6 (*r* = 0.18), suggesting that opinions about water conservation are partially influenced by gender differences combined with education level.

#### Gender and education major

- Gender and education majors demonstrated weaker correlations across most questions, except for Q9 (*r* = 0.16), where biological diversity was perceived as more important among certain groups.

Spearman correlation analyses provided insights into the monotonic relationships between predictors and survey responses. Key findings include:

- Age showed a positive correlation with most questions, indicating that older students are more likely to favor sustainable practices.
- Gender had a moderate correlation with Q6 (*r* = 0.18), suggesting differences in opinions about water conservation.
- Education level exhibited a negative correlation with Q7 (*r* = -0.15), implying that higher education levels may lead to more nuanced views on nature preservation.
- Education majors exhibited consistently significant positive correlations with sustainability-related questions, emphasizing their role in shaping specialized perspectives.

The detailed Spearman correlation coefficients between predictors and survey responses are provided in (Table 10).

**Table 10:** Detailed correlation results.

Predictor	Question	Correlation Coefficient	Direction
Age	Q5	0.25	Positive
Age	Q6	0.30	Positive
Gender	Q6	0.18	Positive
Education Level	Q7	-0.15	Negative
Education Major	Q8	0.22	Positive

### Multinomial logistic regression (MNLogit)

Multinomial logistic regression quantified the effects of demographic predictors on response categories, which represent the levels of agreement or disagreement with the survey questions, ranging from strongly disagree to strongly agree.

Multinomial logistic regression was expanded to include a detailed analysis of each predictor combination for all questions (Q5–Q11). The results for each pair are described below:

#### Age and education level

- **Q5**: Age positively influenced agreement levels (coefficients = 0.1178, *p* < 0.001), while education level had a negative impact (-0.3583, *p* = 0.004).
- **Q6**: Both predictors were significant, with age contributing positively (0.2337, *p* < 0.001) and education level showing negative effects (-0.3523, *p* = 0.007).
- **Q7–Q11**: Significant trends persisted, with higher age favoring agreement and higher education level showing more neutral or disagreeing responses.

#### Age and education major

- **Q5**: Education major strongly influenced positive responses (0.1567, *p* = 0.014).
- **Q6–Q11**: Age and education major consistently contributed significantly, highlighting the combined effect of experience and specialized academic training on sustainability perceptions.

#### Education level and education major

- Across all questions, education major showed a stronger effect than education level, particularly for Q9 (0.4700, *p* < 0.001).

#### Gender and education level

- Gender showed significant interaction effects with education level, particularly for Q6 (coefficients up to 1.9777, *p* = 0.003).

#### Gender and education major

- Gender effects were weaker when paired with an education major, with significance noted only for Q9 (0.2834, *p* = 0.039).
- Regression highlights result that are summarized in Table 11.

**Table 11:** Summary of results.

Predictor Combination	Question	Response category	Coefficient	*p*-Value
Age, Education Level	Q5	2	0.1178,-0.3583	*p* < 0.001, *p* = 0.004
Age, Education Level	Q6	3	0.2337,-0.3523	*P* < 0.001, *P* = 0.007
Education Level, Education Major	Q9	3	0.1799,0.4700	*p* = 0.123, *p* < 0.001
Gender, Education Level	Q6	2	1.2041,0.3363	*P* = 0.003,0.01
Gender, Education Major	Q9	3	0.9530,0.4731	0.121, *p* < 0.001

#### Interpretation of statistical results

##### Age as a predictor of sustainability awareness

The positive correlation between age and sustainability awareness suggests that older students may have:

- Accumulated Life Experiences: Exposure to environmental challenges (e.g., Cairo’s rapid urbanization, pollution) over time may heighten their sensitivity to sustainability issues.
- Longer Educational Exposure: Older students may have completed more courses or extracurricular activities integrating sustainability concepts, even indirectly.
- Developed Responsibility: Maturity and awareness of intergenerational equity (e.g., concerns for future generations) could motivate proactive attitudes.
- Cultural Context: In Egypt, older individuals often hold familial or community leadership roles, which may align with stewardship values embedded in local norms.

##### Discipline-specific differences (education major)

Students in architecture, engineering, or environmental sciences exhibited higher awareness due to:

- Curriculum Integration: These fields often include sustainability modules (e.g., energy-efficient design in architecture, renewable energy systems in engineering).
- Practical Engagement: Hands-on projects (e.g., waste management simulations, green building certifications) foster applied understanding.
- Career Alignment: Majors tied to industries with sustainability mandates (e.g., construction, urban planning) may self-select students already inclined toward environmental stewardship.

##### Gender differences

The gender gap in responses (e.g., Q6 on water conservation) may reflect:

- Sociocultural Roles: Women in Egypt are frequently primary managers of household resources (e.g., water, energy), making them more attuned to conservation practices.
- Educational Priorities: Female students might gravitate toward socially oriented sustainability topics (e.g., community health, equity), aligning with global trends where women prioritize social and environmental dimensions.
- Normative Pressures: Masculine norms in technical fields (e.g., engineering) may emphasize economic growth over environmental preservation, whereas women may challenge such paradigms.

##### Negative association with education level

The counterintuitive finding that higher education levels correlate with lower awareness could stem from:

- Specialization Silos: Advanced students may focus on niche areas lacking sustainability integration (e.g., traditional business curricula ignoring circular economy principles).
- Critical Skepticism: Senior students might question simplistic sustainability narratives (e.g., “recycling solves everything”), leading to neutral/disagree responses on Likert-scale items.
- Curriculum Gaps: Egyptian higher education may prioritize technical skills over holistic sustainability literacy, unlike European models where interdisciplinary SDG-aligned courses are common.

##### Contrasts with global trends

While international studies often link education level to awareness, Egypt’s divergence highlights:

- Structural Shortcomings: Lack of mandatory sustainability courses in non-specialized majors.
- Policy Lag: Despite Egypt Vision 2030, implementation in universities remains uneven, with younger cohorts possibly benefiting more from recent reforms.

##### Recommendations for contextualizing results

- Curriculum Reform: Mandate cross-disciplinary sustainability modules (e.g., ethics in business, eco-design in engineering) to bridge knowledge gaps.
- Gender-Responsive Programs: Leverage female students’ social engagement tendencies through community-led sustainability projects.
- Longitudinal Studies: Track awareness shifts as Egypt Vision 2030 initiatives mature, using mixed methods (e.g., surveys + behavioral observations).
- Cultural Leverage: Integrate Islamic principles of environmental stewardship (Khalifa) into education to resonate with local values.

## Discussion

This study sought to evaluate sustainability awareness among university students in Cairo, Egypt, with a focus on knowledge, behaviors, and attitudes, while accounting for demographic and academic variables such as age, gender, and field of study. The findings offer valuable insights into the current state of sustainability consciousness in the Egyptian higher education context and highlight critical areas for intervention. The results reveal that while a significant portion of students are familiar with the term “sustainability,” there remains a noticeable gap in their practical knowledge, especially in areas such as recycling, renewable energy use, and energy conservation. This highlights the often-cited gap between sustainability awareness and behavioral implementation, a pattern echoed in other regional and international studies. In the current sample, basic familiarity with sustainability concepts did not consistently translate into habitual pro-environmental behavior, underscoring the need to distinguish cognitive awareness from applied action. This aligns with global trends where sustainability awareness often exceeds sustainable behaviors, as documented in studies from the United States, the UK, and parts of Asia^36,53^. However, unlike students in European and North American universities, who often benefit from well-established sustainability curricula and green campus initiatives^38,48^.

The findings of this study affirm and enrich the broader literature on sustainability awareness by highlighting how demographic and academic factors shape students’ sustainability knowledge, attitudes, and behaviors within the Egyptian context. The study’s findings particularly the positive correlation between age and sustainability awareness, gender-based differences, and the influence of academic discipline—can be effectively interpreted through established theoretical frameworks. The Theory of Planned Behavior^62^, helps explain how attitudes shaped by age and gender influence students’ intentions and sustainable behaviors, with older students possibly exhibiting stronger actions due to greater perceived control and normative beliefs. Similarly, Transformative Learning Theory^63^, accounts for the higher awareness among students in sustainability-focused majors, as these disciplines promote critical reflection and perspective shifts. These theoretical integrations provide a deeper understanding of the cognitive and social mechanisms influencing sustainability consciousness, reinforcing the relevance and interpretive depth of our results.

Firstly, age was found to be a significant positive predictor of sustainability attitudes and behaviors among students, supporting previous international findings such as those from Sweden and Germany, where older students demonstrated greater environmental responsibility due to accumulated life experience and cognitive maturity^23,51^. This is also consistent with local research conducted in the Greater Cairo Region. When comparing the results within the local framework, this finding agrees with the conclusion from a study conducted in the Greater Cairo Region (GCR), which demonstrated a substantial level of awareness about two sustainable awareness factors among a sample of approximately 21%, which comprised university students^64^. Similarly, these results are in line with the findings from a survey targeting university students in the GCR and confirm the solid importance of inclusivity as a factor of social sustainability^65^. Additionally, these findings align with the outcomes from a study conducted in Egypt that revealed that age has a significant effect on one of the sustainability behaviors^66^. While people’s perception shapes the sustainability behaviour, our results are supported by the conclusion from a study conducted in Cairo that proved a significant impact of outdoor green spaces on people’s well-being and perception^67^. This reinforces the idea that perceived sustainability values—driven by environmental exposure and personal attitudes—may not always correlate directly with measured behavioral outcomes. Secondly, Gender showed significant effects on certain questions, particularly Q6 and Q8, suggesting that sustainability perspectives vary between male and female respondents, This result aligns with findings from international studies, such as those conducted in Sweden and the United Kingdom, which consistently indicate that female students tend to exhibit stronger pro-environmental attitudes and social responsibility regarding sustainability issues^29,32^. Also, this pattern is consistent with findings from research in Saudi Arabia and other Middle Eastern contexts, where men often focus more on economic and infrastructural sustainability, while women emphasize social and environmental well-being^34^. These results emphasize the need for gender-responsive sustainability education in Egypt that not only promotes inclusivity but also addresses the different orientations and strengths that male and female students bring to sustainability learning. The variation in gender responses supports previous work that encourages the development of curricula that balance technical knowledge with social engagement to foster holistic sustainability awareness^32^. Thirdly, while international literature generally reports a positive correlation between education level and sustainability knowledge^35,53^, our study found a negative association. This divergence may indicate gaps in the sustainability integration within Egyptian higher education programs, particularly in non-specialized fields. However, the increasing sustainability awareness among younger students suggests that recent educational reforms, including those under Egypt Vision 2030, may be taking root and benefiting new cohorts. Lastly, the positive influence of a student’s education major aligns with numerous international findings. Studies from the United Kingdom and Sweden have demonstrated that students in programs such as architecture, engineering, and environmental sciences exhibit higher sustainability awareness due to more focused exposure^42,43^. This confirms that discipline-specific curricula play a vital role in fostering sustainability values and practices. Overall, while several findings are in harmony with international trends, the distinct patterns observed in Egypt—particularly regarding education level—highlight the need for more targeted curriculum development and policy interventions in higher education.

These findings echo global research that emphasizes the complex relationship between environmental awareness and sustainable behavior. For instance, in^68^, a significant positive correlation was found between environmental consciousness and proactive actions among engineering professionals, yet the study also acknowledged that awareness alone is often insufficient without reinforcement through values, norms, and contextual factors. Similarly, research conducted in Peru showed that environmental awareness significantly influenced consumers’ willingness to pay for green products, with the Theory of Planned Behavior highlighting the roles of attitude and perceived behavioral control^69^. In another study conducted in the United Arab Emirates, environmental awareness was found to directly affect both attitudes and purchase intentions toward eco-friendly products, though not necessarily mediated by attitude alone^70^. These comparative examples reinforce the current study’s finding that while sustainability awareness among students in Cairo is present, it does not always translate into consistent behavioral change. Thus, behavioral engagement must be cultivated alongside awareness through structured, supportive educational and policy frameworks.

These findings emphasize the urgent need for Egypt’s educational policymakers to mandate sustainability education across all disciplines, support female-led and discipline-specific initiatives, and engage younger cohorts through practical learning. However, it is essential to note that awareness alone may not be sufficient to foster sustained behavior without deeper integration into lived experiences and institutional structures. Therefore, future interventions should include behavior-based approaches—such as hands-on sustainability projects, gamified competitions, and community outreach programs—to reinforce actionable learning and long-term habit formation. Integrating sustainability into university curricula, backed by Egypt Vision 2030, and fostering collaboration between ministries, educators, and municipalities will be essential for developing a generation capable of informed sustainable action.

While the study provides valuable insights into sustainability awareness among university-level students in Cairo, it is limited by its reliance on self-reported questionnaire data, which may not fully capture actual behaviors or deeper conceptual understanding. Additionally, the sample, though numerically sufficient, may not reflect the full diversity of institutional types, academic disciplines, or geographic variations across the broader Cairo region. Additionally, the study’s statistical analysis limitations are highlighted in the potential non-linearity, the sparse categories, the model fit, and the experience of missing data.

## Conclusion

This study assesses the level of sustainability consciousness among university students in Cairo, Egypt, emphasizing the influence of academic levels and socio-educational backgrounds on their viewpoints. A questionnaire was distributed to several schools, resulting in 524 replies over one year. The data was examined using several statistical techniques, including chi-squared testing, Spearman correlation, and multinomial logistic regression. The study investigated the relationship between sustainability awareness and socio-educational factors, producing the following main results:

- **Age as a predictor**: Age consistently emerged as a significant positive predictor across all response categories, indicating that older students tend to exhibit more developed sustainability attitudes and behaviors.
- **Gender differences**: Gender showed significant effects on certain questions, particularly Q6 and Q8, suggesting that sustainability perspectives vary between male and female respondents.
- **Education level and major**: The results highlight a complex relationship—while higher education levels showed negative associations with sustainability awareness, the education major had a positive influence, underscoring the distinct impacts of general versus specialized knowledge.

These results underline the need to include demographic factors in the analysis of responses on sustainability-related issues. The continuous influence of age implies that perceptions of sustainability are shaped in great part by life stage and experience. Moreover, the different effects of the degree of education and major show the need for a more sophisticated strategy when including sustainability issues in courses.

The observed negative association between education level and sustainability awareness, in contrast to international findings that typically show a positive correlation, points to potential gaps in how sustainability is embedded in Egyptian higher education, particularly in general or non-specialized programs. However, the relatively higher awareness among younger students may reflect the early impacts of recent educational reforms, such as those outlined in Egypt Vision 2030, suggesting progress toward cultivating a more sustainability-conscious student population in the coming years.

The study’s limitations stem from the reliance on self-reported data, which may introduce biases. Future research could broaden the methodological scope by incorporating longitudinal studies to track shifts in attitudes over time or by integrating qualitative insights to complement the quantitative findings. This research enhances our understanding of how demographic factors influence sustainability awareness and highlights the need for strategies that bridge the gap between knowledge and consistent behavioral engagement. The findings offer actionable insights for educators and policymakers aiming to design targeted awareness initiatives that resonate with diverse student populations.

## Electronic supplementary material

Below is the link to the electronic supplementary material.

Supplementary Material 1

## Data Availability

Datasets used and/or analyzed during the current study are available from the corresponding author on reasonable request.
