# Supplementary material for: Sustainability awareness assessment for university-level students in Cairo, Egypt
Source: Sci Rep. 2026 Feb 20;16:7723. doi: 10.1038/s41598-025-08575-1 (PMC12948997; doi:10.1038/s41598-025-08575-1)
Supplement: Supplementary file 1 — Supplementary Material 1 [file 41598_2025_8575_MOESM1_ESM.docx]

## Appendix A

(Frequency Table)

Table S1 Frequency distribution of demographic and response variables

*Legend: This supplementary table presents a detailed frequency summary of participants' demographic characteristics and survey responses. It offers a comprehensive overview of the dataset and supports the interpretation of the statistical analyses discussed in the main text.*

| Education Level | Education Major | Frequency |
| --- | --- | --- |
| Bachelor Degree | Accounting | 1 |
| Bachelor Degree | Architecture Engineering | 7 |
| Bachelor Degree | Business Administration | 1 |
| Bachelor Degree | a | 1 |
| Bachelor Degree | Civil Engineering | 4 |
| Bachelor Degree | Computer Engineering | 2 |
| Bachelor Degree | Computer Science | 2 |
| Bachelor Degree | Construction Management | 1 |
| Bachelor Degree | Electronics | 1 |
| Bachelor Degree | Faculty of Dentistry | 1 |
| Bachelor Degree | Faculty of Law | 1 |
| Bachelor Degree | Faculty of Mass Communication | 1 |
| Bachelor Degree | Faculty of Medicine | 1 |
| Bachelor Degree | Finance | 1 |
| Bachelor Degree | Graphic Design | 1 |
| Bachelor Degree | History | 1 |
| Bachelor Degree | Human Medicine | 1 |
| Bachelor Degree | Information Engineering and Technology | 1 |
| Bachelor Degree | Information System | 1 |
| Bachelor Degree | Marketing | 1 |
| Bachelor Degree | Media Engineering and Technology | 1 |
| Bachelor Degree | Medicine and Surgery | 1 |
| Bachelor Degree | Political Science | 1 |
| Bachelor Degree | Structural Civil Engineering | 1 |
| Level 1 | Accounting and Business Administration | 39 |
| Level 1 | Al Alsun | 1 |
| Level 1 | Applied Arts | 5 |
| Level 1 | BIS | 1 |
| Level 1 | Business | 1 |
| Level 1 | Business Francais | 1 |
| Level 1 | Computer Science | 1 |
| Level 1 | Computer and Artificial Intelligence | 1 |
| Level 1 | Education | 1 |
| Level 1 | Electrical Engineering | 1 |
| Level 1 | Electronics Engineering | 1 |
| Level 1 | Faculty of Arts | 2 |
| Level 1 | Faculty of Commerce | 17 |
| Level 1 | Faculty of Dentistry | 2 |
| Level 1 | Faculty of Engineering and Technology | 11 |
| Level 1 | Faculty of Law | 3 |
| Level 1 | Faculty of Mass Communication | 1 |
| Level 1 | Faculty of Medicine | 3 |
| Level 1 | Faculty of Pharmacy | 2 |
| Level 1 | Faculty of Science | 2 |
| Level 1 | HR | 1 |
| Level 1 | Informatics | 7 |
| Level 1 | Management | 1 |
| Level 1 | Marketing and Foreign Trade | 1 |
| Level 1 | Marketing and Management | 2 |
| Level 1 | Mechanical Engineering | 1 |
| Level 1 | Media | 3 |
| Level 1 | Nursing | 2 |
| Level 1 | Physical Therapy | 2 |
| Level 1 | Political Science | 1 |
| Level 1 | Psychology | 1 |
| Level 1 | Trade and Business Administration | 1 |
| Level 2 | Accounting | 2 |
| Level 2 | Accounting and Business Administration | 6 |
| Level 2 | Applied Arts | 3 |
| Level 2 | BIS | 1 |
| Level 2 | Biomedical Engineering | 2 |
| Level 2 | Broadcasting | 1 |
| Level 2 | Cinematography | 1 |
| Level 2 | Civil Engineering | 4 |
| Level 2 | Clothing Department | 1 |
| Level 2 | Computer | 25 |
| Level 2 | Computer and Systems Engineering | 1 |
| Level 2 | Construction | 1 |
| Level 2 | Economic Studies | 1 |
| Level 2 | Economics and Political Science | 1 |
| Level 2 | Education | 2 |
| Level 2 | Electrical Engineering | 3 |
| Level 2 | Environmental Sustainable Architecture | 1 |
| Level 2 | Faculty of Agriculture | 1 |
| Level 2 | Faculty of Arts | 1 |
| Level 2 | Faculty of Commerce | 9 |
| Level 2 | Faculty of Dentistry | 3 |
| Level 2 | Faculty of Mass Communication | 4 |
| Level 2 | Faculty of Medicine | 3 |
| Level 2 | Faculty of Science | 3 |
| Level 2 | Fashion | 1 |
| Level 2 | Finance | 2 |
| Level 2 | Fine Arts | 1 |
| Level 2 | General Trading | 1 |
| Level 2 | History | 1 |
| Level 2 | Informatics | 2 |
| Level 2 | Interior Designing | 3 |
| Level 2 | Law and Legal Studies | 1 |
| Level 2 | Marketing | 2 |
| Level 2 | Mass Communication | 1 |
| Level 2 | Mechanical Engineering | 1 |
| Level 2 | Mechatronics Engineering | 1 |
| Level 2 | Media Production Center | 1 |
| Level 2 | Medical | 1 |
| Level 2 | Nursing | 1 |
| Level 2 | Nutrition Sciences | 1 |
| Level 2 | Photography Department | 2 |
| Level 2 | Political Science | 3 |
| Level 2 | Printing and Publishing | 1 |
| Level 2 | Social Service | 1 |
| Level 2 | Software Engineering | 1 |
| Level 2 | Trade and Business Administration | 1 |
| Level 3 | Accounting | 4 |
| Level 3 | Actuarian Studies | 1 |
| Level 3 | Administration | 1 |
| Level 3 | Applied Arts | 1 |
| Level 3 | Architectural Sculpture and Formation | 2 |
| Level 3 | Architecture Engineering | 16 |
| Level 3 | Business | 1 |
| Level 3 | Civil Engineering | 17 |
| Level 3 | Communication and Computer Engineering | 1 |
| Level 3 | Computer | 8 |
| Level 3 | Computer Science | 2 |
| Level 3 | Dentistry | 1 |
| Level 3 | Electrical Engineering | 7 |
| Level 3 | Electronic Media | 1 |
| Level 3 | Energy and Renewable Energy | 1 |
| Level 3 | Entrepreneurship | 1 |
| Level 3 | Environmental Sustainable Architecture | 1 |
| Level 3 | Faculty of Commerce | 4 |
| Level 3 | Faculty of Dentistry | 3 |
| Level 3 | Faculty of Mass Communication | 4 |
| Level 3 | Faculty of Medicine | 2 |
| Level 3 | Faculty of Pharmacy | 1 |
| Level 3 | Faculty of Science | 6 |
| Level 3 | Fashion | 5 |
| Level 3 | Finance | 2 |
| Level 3 | General Administration | 1 |
| Level 3 | General Trading | 1 |
| Level 3 | Geography | 1 |
| Level 3 | Information Technology | 1 |
| Level 3 | Institute of Social Service | 1 |
| Level 3 | Interior Designing | 12 |
| Level 3 | Law | 1 |
| Level 3 | Management | 1 |
| Level 3 | Marketing | 2 |
| Level 3 | Mechanical Engineering | 3 |
| Level 3 | Mechatronics | 1 |
| Level 3 | Media | 1 |
| Level 3 | Medicines and Poison | 1 |
| Level 3 | Pharmaceutical Sciences | 1 |
| Level 3 | Photography | 3 |
| Level 3 | Physical Therapy | 2 |
| Level 3 | Physiotherapy | 1 |
| Level 3 | Political Science | 1 |
| Level 3 | Printing and Publishing | 1 |
| Level 3 | Ready-made Clothing Department | 2 |
| Level 3 | Special International Law | 1 |
| Level 3 | Technology | 4 |
| Level 3 | Tourist Guides | 1 |
| Level 3 | Translation | 1 |
| Level 3 | Urban Planning | 1 |
| Level 4 | Accounting and Business Administration | 1 |
| Level 4 | Applied Arts | 1 |
| Level 4 | Architecture Engineering | 22 |
| Level 4 | BIS | 1 |
| Level 4 | Bio | 1 |
| Level 4 | Business Administration | 1 |
| Level 4 | Business Informatics | 1 |
| Level 4 | Civil Engineering | 4 |
| Level 4 | Computer | 2 |
| Level 4 | Computer Science | 2 |
| Level 4 | Faculty of Commerce | 1 |
| Level 4 | Faculty of Dentistry | 1 |
| Level 4 | Faculty of Law | 1 |
| Level 4 | Faculty of Mass Communication | 3 |
| Level 4 | Faculty of Medicine | 1 |
| Level 4 | Faculty of Science | 1 |
| Level 4 | Finance | 3 |
| Level 4 | Industrial Design | 1 |
| Level 4 | MET | 1 |
| Level 4 | MPharm | 1 |
| Level 4 | Management | 1 |
| Level 4 | Marketing and Management | 2 |
| Level 4 | Mechatronics | 1 |
| Level 4 | Petroleum Engineering | 1 |
| Level 4 | Physical Therapy | 1 |
| Level 4 | Plant Production | 1 |
| Level 4 | Political Science | 1 |
| Level 4 | Production | 1 |
| Level 4 | Public International Law | 1 |
| Level 4 | Public Law | 1 |
| Level 4 | Radio and TV | 1 |
| Level 4 | Sculpting and Shaping | 3 |
| Level 4 | Technology | 1 |
| Level 4 | Urban Planning | 1 |
| Level 6 | Faculty of Medicine | 1 |
| Masters | Information Engineering and Technology | 1 |
| Masters Degree | Accounting and Finance | 1 |
| Officer | Artillery | 1 |
| PhD | Architecture Engineering | 3 |
| PhD | Civil Engineering | 1 |
| PhD | Mathematics | 1 |
| Post Graduate | Business | 1 |
| Post Graduate | Management | 1 |
| Senior | Architecture Engineering | 15 |
| Senior | Civil Engineering | 9 |
| Senior | Clinical | 1 |
| Senior | Construction Engineering | 1 |
| Senior | Cyber Security | 1 |
| Senior | Dental Medicine | 1 |
| Senior | Faculty of Dentistry | 3 |
| Senior | Faculty of Law | 2 |
| Senior | German | 1 |
| Senior | Graphic | 1 |
| Senior | Mechanical Engineering | 1 |
| Senior | Mechatronics | 1 |
| Senior | Petroleum Engineering | 2 |
| Senior | Photography Department | 1 |
| Senior | Project Management | 1 |
| Senior | Support and Rehabilitation Sciences | 1 |
| Senior | Veterinarian | 1 |
| Total Data |  | total=524 |

## Appendix B

(Questionnaire)

Dear Respondent,
 We are an interdisciplinary research group that is currently conducting academic research on “Sustainability awareness assessment for university-level students in Cairo, Egypt.”. This research targets academic students who are currently registered in universities in Egypt (private, public, or both). We are inviting you to participate in this research study by completing this questionnaire.

The following questionnaire will require approximately less than 5 minutes to complete. There is no compensation for responding, nor is there any known risk. To ensure that all information remains confidential, these survey answers will be used for study purposes only. If you choose to participate in this project, please answer all questions honestly.

Thank you for taking the time to assist us in our educational endeavors. The data collected will provide helpful information regarding the study. If you require additional information or have questions, please contact us.

Yours sincerely,
Research Team

Demographic Data

- Age
- Gender
- High school
- University name
- Faculty name
- Education Level
- Education Major

**Sustainability knowingness**

Question 1: I am familiar with the meaning of the word “sustainability”.

| **4**  **Strongly Agree** | **3**  **Agree** | **2**  **Disagree** | **1**  **Strongly Disagree** |
| --- | --- | --- | --- |

Question 2: Your source of sustainability information.

| **5**  **High school** | **4**  **university** | **3**  **Public lectures** | **2**  **Social media** | **1**  **Other (please state)** |
| --- | --- | --- | --- | --- |

Question 3: The number of subjects related to the environment and sustainability that you studied in your faculty as part of your curriculum content.

| **5**  **None** | **4**  **0-2** | **3**  **2-4** | **2**  **4-6** | **1**  **More than6** |
| --- | --- | --- | --- | --- |

Question 4: The number of sustainability-related activities in your university.

| **5**  **None** | **4**  **0-2** | **3**  **2-4** | **2**  **4-6** | **1**  **More than 6** |
| --- | --- | --- | --- | --- |

Question 5: The implementation of sustainable actions on the university campus will improve

the physical conditions within it.

| **4**  **Strongly Agree** | **3**  **Agree** | **2**  **Disagree** | **1**  **Strongly Disagree** |
| --- | --- | --- | --- |

Question 6: Water conservation is essential for sustainable development.

| **4**  **Strongly Agree** | **3**  **Agree** | **2**  **Disagree** | **1**  **Strongly Disagree** |
| --- | --- | --- | --- |

Question 7: Nature preservation is not required for sustainable development.

| **4**  **Strongly Agree** | **3**  **Agree** | **2**  **Disagree** | **1**  **Strongly Disagree** |
| --- | --- | --- | --- |

Question 8: Humans must reduce all types of waste to achieve sustainable development.

| **4**  **Strongly Agree** | **3**  **Agree** | **2**  **Disagree** | **1**  **Strongly Disagree** |
| --- | --- | --- | --- |

Question 9: The preservation of the diversity of living creatures (biological diversity) is required for sustainable development.

| **4**  **Strongly Agree** | **3**  **Agree** | **2**  **Disagree** | **1**  **Strongly Disagree** |
| --- | --- | --- | --- |

Question 10: A shift to natural renewable natural resources is required for sustainable development.

| **4**  **Strongly Agree** | **3**  **Agree** | **2**  **Disagree** | **1**  **Strongly Disagree** |
| --- | --- | --- | --- |

Question 11: People must be educated on how to protect themselves against natural disasters in order to achieve sustainable development.

| **4**  **Strongly Agree** | **3**  **Agree** | **2**  **Disagree** | **1**  **Strongly Disagree** |
| --- | --- | --- | --- |

**Appendix C**

(Code Snippets)

# Reverse scoring implementation
response_mapping = {'strongly agree': 4, 'agree': 3, 'disagree': 2, 'strongly disagree': 1}
reverse_mapping = {4:1, 3:2, 2:3, 1:4}
df['Q7'] = df['Q7'].str.lower().str.strip().map(response_mapping).map(reverse_mapping).fillna(0)

----------------------------------------------------------------------------------------------------------

# Odds Ratio and CI calculation
import statsmodels.api as sm
model = sm.MNLogit(y, X).fit()
params = model.params
cis = model.conf_int()
odds_ratios = pd.DataFrame({
 'OR': np.exp(params),
 'CI_lower': np.exp(cis[:, 0]),
 'CI_upper': np.exp(cis[:, 1])
})

--------------------------------------------------------------------------------------------------------------

# Regularized multinomial regression
model = sm.MNLogit(y_train, X_train).fit_regularized(alpha=0.001, maxiter=5000, tol=1e-6, method='l1_cvxopt_cp')
-------------------------------------------------------------------------------------------------------------

# Calculate VIF scores
from statsmodels.stats.outliers_influence import variance_inflation_factor
def calculate_vif(df, predictors):
 X = df[predictors]
 X = sm.add_constant(X)
 vif = pd.DataFrame()
 vif['Variable'] = X.columns
 vif['VIF'] = [variance_inflation_factor(X.values, i) for i in range(X.shape[1])]
 return vif
